# Maintenance of Gut Microbial Balance via the Kynurenine Pathway Improves Larval Performance and Resistance to *Bacillus thuringiensis* in *Spodoptera exigua*

**DOI:** 10.1002/mbo3.70289

**Published:** 2026-04-16

**Authors:** Daniel Pinos, Elena García‐Marín, Beatriz Ramírez‐Serrano, Luis Benavent‐Albarracín, Jordi Gamir, Cristina M. Crava

**Affiliations:** ^1^ Instituto Universitario de Biotecnología y Biomedicina (BIOTECMED), Universitat de València Burjassot Spain; ^2^ Department of Biology, Biochemistry and Environmental Sciences Plant Immunology and Biochemistry Group, Universitat Jaume I, Castellón de la Plana Spain

**Keywords:** 8‐hydroxyquinoline‐2‐carboxylic acid, CRISPR/Cas9, *Enterococcus*, kynurenine 3‐monooxygenase, tryptophan metabolism

## Abstract

The gut microbiota is a key determinant of insect physiology, influencing nutrition, immunity, and interactions with plants and pathogens. In Lepidoptera, larval gut communities are dynamic, but a core microbiota, often dominated by *Enterococcus* species, persists across instars. In *Spodoptera littoralis*, the enzyme kynurenine 3‐monooxygenase (KMO) regulates gut bacterial composition via 8‐hydroxyquinoline‐2‐carboxylic acid (8‐HQA), a secreted iron‐chelating compound. To investigate whether this mechanism is conserved in Noctuidae, we generated *Spodoptera exigua kmo* ^*‐/‐*^ mutants using CRISPR/Cas9 and analyzed bacterial communities in foregut, midgut, hindgut, and oral secretions by 16S metabarcoding, using RNA‐derived cDNA for gut samples and DNA for oral secretions due to lower microbial biomass. The *kmo* deletion abolished 8‐HQA production, reduced bacterial diversity, and collapsed compartment‐specific bacterial communities in the gut, while also being associated with *Enterococcus* dominance in oral secretions. Fitness assays revealed that *kmo* ^‐/‐^ larvae exhibited reduced weight gain on artificial diet, and higher mortality and delayed growth when fed on pepper leaves. Moreover, *kmo* ^*‐/‐*^ larvae were threefold more susceptible to *Bacillus thuringiensis*, consistent with an interaction between host physiological state, gut microbial homeostasis, and pathogen susceptibility. Dietary supplementation with 8‐HQA partially mitigated, but did not fully rescue, growth deficits. Our results demonstrate that the kynurenine pathway and 8‐HQA production are crucial for maintaining gut microbial homeostasis, particularly within *Enterococcus*, thereby supporting larval development, dietary adaptation, and pathogen resilience. These findings reveal a conserved mechanism in noctuid moths linking host metabolism, microbiota regulation, and ecological performance, emphasizing the interplay between host genetics, microbiota composition, and environmental stressors.

## Graphical abstract

CRISPR/Cas9‐mediated deletion of *kmo* in *Spodoptera exigua* abolished 8‐HQA production, disrupted gut microbial homeostasis, and promoted *Enterococcus* dominance in gut and oral secretion. Mutants exhibited impaired growth, delayed development, and increased susceptibility to *Bacillus thuringiensis*, highlighting a conserved role of the kynurenine pathway in linking host metabolism, microbiota regulation, and larval fitness.

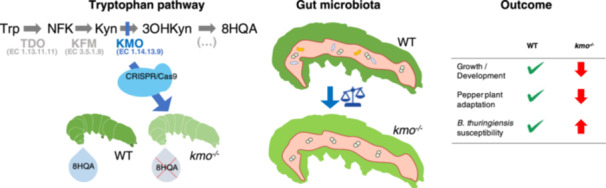

## 1 Introduction

The gut microbiota is considered an integral component of the host phenotype, influencing key biological traits such as immunity, nutrition, and behavior (Engel and Moran 2013; Shao et al. 2024). In insects, the microbiota can also shape trophic interactions with host plants by producing toxin‐degrading enzymes or modulating plant defense responses (Mason et al. 2019). Moreover, it may interact with entomopathogens or natural enemies ‐offering protection or immunity the host—and serve as a source of enzymes capable of detoxifying chemical insecticides (Mason 2020; Shao et al. 2024).

In holometabolous insects, such as Lepidoptera, microbiota undergo dramatic changes after metamorphosis (Manthey et al. 2023). During the larval stage, the digestive tract of lepidopterans is a simple tubular structure composed of three main segments: the foregut, midgut, and hindgut (Wu et al. 2016). The foregut is responsible for food intake, mechanical processing, and temporary storage; the midgut serves as the principal site for digestion and nutrient absorption; and the hindgut functions in reabsorbing water and nutrients before excretion of waste (Engel and Moran 2013; Wu et al. 2016). Due to this continuous passage of food and the absence of specialized gut structures, researchers long suggested that Lepidoptera larvae lack a stable core microbiota and instead harbor only transient microbes (Hammer et al. 2017). This view is largely driven by substantial differences observed in metabarcoding studies, which revealed strikingly divergent microbiota profiles between laboratory‐reared colonies—often fed artificial diets—and field‐collected individuals (Broderick et al. 2004; Martínez‐Solís et al. 2020; Gayatri Priya et al. 2012). However, growing evidence suggests that larval digestive systems are not passive carriers of microbes. Despite variation in gut microbial composition, recent studies point toward the existence of a core microbiota, typically dominated by a few persistent bacterial species and accompanied by a large number of transient taxa (Higuita Palacio et al. 2021; Mason 2020; Oliveira et al. 2023; Gayatri Priya et al. 2012; Tang et al. 2012; Teh et al. 2016). For instance, in Noctuidae, a substantial portion of the gut microbiota is composed of gram‐positive Firmicutes (~ 25%) and gram‐negative Proteobacteria (~ 55%). While many Proteobacteria occur in the plant phyllosphere, indicating a possible diet‐associated origin, Firmicutes are less commonly found on plants, suggesting selective filtering by the host (Shao et al. 2024).

Within Firmicutes, emerging evidence highlights the relevance of *Enterococcus* in noctuid larvae. In *Spodoptera littoralis*, experiments using GFP‐tagged strains have shown that *Enterococcus mundtii* persists in the larval gut across successive molts, suggesting a stable association with the host (Teh et al. 2016). In *Spodoptera frugiperda*, gut‐associated *Enterococcus* species modulate larval growth in a diet‐dependent manner, facilitating development under nutrient‐poor conditions, thus playing a key role in shaping host performance (Fu et al. 2025; Mason et al. 2020). Beyond Noctuidae, *Enterococcus* species have also been implicated in immune modulation and developmental processes. In the crambid *Conogethes punctiferalis*, *E. mundtii* promotes the growth of early‐instar larvae (J. Li et al. 2024). In *Galleria mellonella* (Pyralidae) *Enterococcus* appears to stimulate gut immune responses and confers protection against *Bacillus thuringiensis* infection (Upfold et al. 2023). Additionally, in this species, elimination of *Enterococcus* from the gut microbiota accelerates the larval‐to‐pupal transition (Kong et al. 2023).

The maintenance of such core microbiota is molded by many biotic and abiotic factors. While diet and environment are the primary sources of microbial acquisition—and newly encountered microbes may supplant initial colonizers as larvae feed on different plants—host physiology may also play a central role in shaping microbiota composition, as observed in vertebrates (Reese and Dunn 2018). However, the host physiological mechanisms influencing microbial diversity in Lepidopteran larvae remain poorly understood. Recent work in *S. littoralis* has highlighted the role of the enzyme kynurenine 3‐monooxygenase (KMO), in regulating gut microbiota composition through the production of 8‐hydroxyquinoline‐2‐carboxylic acid (8‐HQA), a compound excreted into the gut lumen. 8‐HQA, an iron‐chelating molecule, is produced in the midgut of Noctuidae larvae and is highly abundant in oral secretions (Pesek et al. 2014). In *S. littoralis*, loss of 8‐HQA results in increased bacterial load and reduced bacterial diversity in the gut, suggesting a major regulatory function (Mazumdar et al. 2023). It is synthesized from tryptophan via kynurenine and 3‐hydroxykynurenine, which is the product of the KMO enzymatic reaction. The kynurenine pathway is responsible for over 95% of oxidative tryptophan degradation in animals and is well studied in insects for its role in the ommochrome biosynthesis pathway that generates eye pigmentation. Mutations in *kmo* have frequently been used as transformation markers in gene‐editing protocols (Q. Han et al. 2003; W.‐K. Han et al. 2023; Hong et al. 2020; Lorenzen et al. 2002; Matsuda et al. 2024; Mazumdar et al. 2023; Purusothaman et al. 2021; Quan et al. 2002; Xu et al. 2020). However, findings in *S. littoralis* also suggest an important role for the kynurenine pathway in shaping the larval microbiota.

In this study, we tested whether this effect is conserved across Noctuidae by targeting KMO in *Spodoptera exigua* via CRISPR/Cas9. To this end, we generated a CRISPR/Cas9 mutant colony with a targeted deletion in the *kmo* gene. We confirmed that the mutation abolishes 8‐HQA production in larval oral secretion and subsequently analyzed bacterial communities by 16S metabarcoding, focusing on transcriptionally active bacteria in gut sections and total bacterial DNA in oral secretions. Special care was taken to separately analyze the three gut segments (foregut, midgut, and hindgut), as well as the bacterial content in the oral secretions, which derive from regurgitated gut contents and have been shown to influence host‐plant interactions (Acevedo et al. 2017; Mason et al. 2019; Pan et al. 2023; Yamasaki et al. 2021). We assessed whether *kmo* deletion affects larval growth on artificial diet and pepper leaves and modulates susceptibility to *B. thuringiensis*. Overall, our results highlight a crucial role of the kynurenine pathway in preserving larval fitness under natural conditions, likely via maintenance of gut microbiota homeostasis.

## 2 Materials and Methods

### 2.1 Insects and Rearing Conditions

A *S. exigua* laboratory colony originally founded from eggs provided by Andermatt Biocontrol AG (Grossdietwil, Switzerland) and maintained for more than 100 generations at the University of Valencia (Spain) was used as the source for the individuals edited by CRISPR/Cas9. To ensure comparable rearing conditions with the edited individuals (*kmo* ^‐^/^‐^), we established a sub‐colony (hereafter referred to as wild type), which was maintained under the same conditions and in the same incubator as the mutant colony. Incubator conditions were: 25 ± 3°C, 60 ± 5% of relative humidity and a 16 h light:8 h dark photoperiod. Both wild‐type and *kmo* ^‐^/^‐^ larvae were fed an antibiotic‐free artificial diet during the larval stage (Elvira et al. 2010). Adults were provided ad libitum with a 10% (w/v) sugar solution.

### 2.2 Generation of CRISPR/Cas9 Mutants

**Figure 1:**
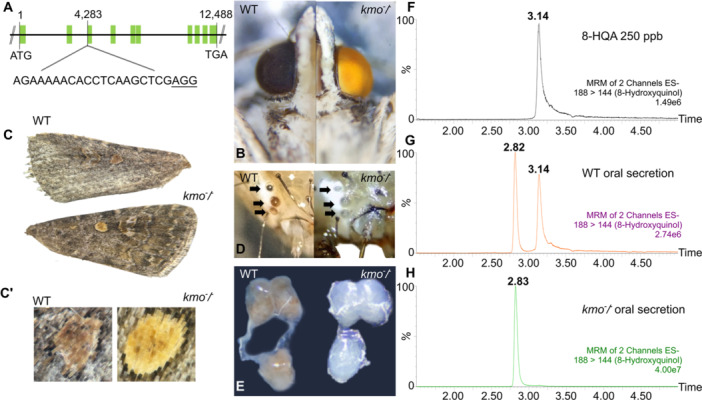
CRISPR/Cas9‐induced mutations in *kmo* and associated phenotypic changes. (A) Schematic representation of the *S. exigua kmo* gene and the target gRNA. Green boxes indicate exons; the PAM sequence is underlined. (B) Eye color change in adult moths, from black in wild‐type (WT) to bright yellow in *kmo* ^⁻/⁻^. (C) Wing pigmentation change in adults, from dark brown in WT to golden yellow in *kmo* ^⁻/⁻^. (C**'**) Detail of wing spots (D), ocelli and (E) brain coloration change in larvae, from dark brown in WT to transparent white in *kmo* ^⁻/⁻^. Chromatograms of (F) 8‐hydroxyquinoline‐2‐carboxylic acid standard; (G) oral secretion from WT larvae; (H) oral secretion from *kmo* ^⁻/⁻^mutants.

The *S. exigua* gene encoding kynurenine 3‐monooxygenase (KMO) was identified by a BlastP analysis using the *S. frugiperda* KMO amino acid sequence (Bioinformatics Platform for Agroecosystem Arthropods, acc. num. GSSPFG00006872001.3‐PA) as query against the BLAST non‐redundant database restricted to *S. exigua*. The gene encoding the best hit (unnamed protein product, acc. num. CAH0701961) comprised 10 exons; the third exon was chosen as the target site for editing. gRNA was designed using the “Find CRISPR sites” tool in Geneious v 10.2.6, considering both on‐target and off‐target activity scores, with the *kmo* gene and the *S. exigua* genome (GCA_902829305.4) as a reference (Figure 1A). crRNA corresponding to the functional RNA molecules carrying the gRNA target sequence was synthesized by IDT DNA. The ribonucleoprotein complex preparation was prepared by annealing Alt‐R CRISPR‐Cas9 crRNA and tracrRNA according to the manufacturer's instructions (IDT DNA). The complex was diluted in 5 mM KCl, 0.1 M sodium phosphate (pH 6.8) and mixed in a ratio 1:1 with Alt‐R Sp Cas9 Nuclease V3 (IDT DNA) to obtain the injection mix.

Eggs less than 1 h post‐oviposition were collected, surface sterilized by immersion with 0.1% sodium hypochlorite for 1 min, rinsed with dH_2_O and injected using a home‐made pneumatic‐operated microinjector (kindly gifted by Prof. D. G. Heckel). Injection needles were prepared from borosilicate capillaries with filament (1.0 × 0.78 × 100 mm) using a Sutter P‐97 micropipette puller (Sutter Instruments, Novato, CA). Injected eggs were incubated in Petri dishes under controlled environmental conditions until hatching.

From G_0_ surviving adults, 9 moths with yellow to orange eyes were intercrossed to generate the F_1_ generation. DNA from dead G_0_ dead adults was extracted from whole heads using DNAZol reagent (Invitrogen) following the manufacturer's instructions. In F_1_ larvae, mutations were screened in the fourth instar by collecting haemolymph through a pseudo‐leg puncture. DNA was extracted by incubating the haemolymph with 5% Chelex 100 Resin (Bio‐Rad) for 1 h at 58°C with agitation, followed by a 15 min incubation at 96°C. After centrifugation (6000 x*g* for 30 s), the supernatant was used as template DNA.

PCR reactions were carried out in a 25 μL volume containing 0.25 μM of forward (ATGAACATCCGCAACAACCGAG) and reverse (CTTGTTCAGAAAACTACTCATAAAAGGCA) primers, 1 μL DNA template, 12.5 μL NZYTaq II 2x Green Master Mix (NZYTech) and 9.5 μL ddH2O. The cycling program included an initial denaturation of 1 min at 95°C, followed by 35 cycles of 95°C for 30 s, 58°C for 30 s and 72°C for 1 min, with a final extension at 72°C for 5 min. Amplification products were visualized on 1% agarose gels, purified with NucleoSpin Gel & PCR Clean‐up mini kit (Macherey‐Nagel, Düren, Germany), and Sanger‐sequenced at STAB‐vida facilities (Portugal). Sequences were analyzed with Geneious (v 10.2.6) software. Once gene editing in mutant individuals was confirmed, the *kmo* ^‐^/^‐^ colony was backcrossed and maintained across generations by visual identification of the yellow‐eye phenotype in adult moths.

### 2.3 8‐HQA Detection in Caterpillar Oral Secretion

Oral secretion (OS) was collected from fifth‐instar larvae of wild‐type and *kmo* ^‐^/^‐^ colonies using a 10 μL pipette, while gently massaging the top of the larval head to stimulate regurgitation. OS from 10 individuals was pooled and immediately frozen at –80°C. Detection of 8‐HQA was performed using liquid chromatography‐electrospray ionization tandem mass spectrometry (LC‐ESI‐MS/MS). To optimize MS parameters, direct infusion of a commercial 8‐HQA standard (CAS No. 148‐24‐3, Sigma‐Aldrich) was used to determine the most abundant product ion. The selected mass transition for single reaction monitoring (SRM) was *m*/*z* 188 → 144, with a collision energy of 10 eV (Figure 1F). OS samples were diluted 1:100 in H₂O:MeOH (90:10) containing 0.01% formic acid (HCOOH), and 5 μL of the diluted sample was injected into an Acquity UPLC system (Waters, Milford, MA, USA) coupled to a triple quadrupole mass spectrometer (TQS; Waters, Manchester, UK). Chromatographic separation was performed using a Kinetex Core‐Shell C18 analytical column (50 × 2.1 mm, 2.6 μm particle size; Phenomenex). The mobile phases were: (A) H₂O + 0.01% HCOOH and (B) MeOH + 0.01% HCOOH. The gradient started at 90% A, decreased linearly to 10% A over 3 min, at isocratic conditions for 2 min, returned to initial conditions within 1 min, and equilibrated for 2 min, with a total run time of 8 min. The flow rate was maintained at 0.3 mL min⁻¹.

### 2.4 Larval Gut and Oral Secretion 16s Metabarcoding Analysis

**Figure 2:**
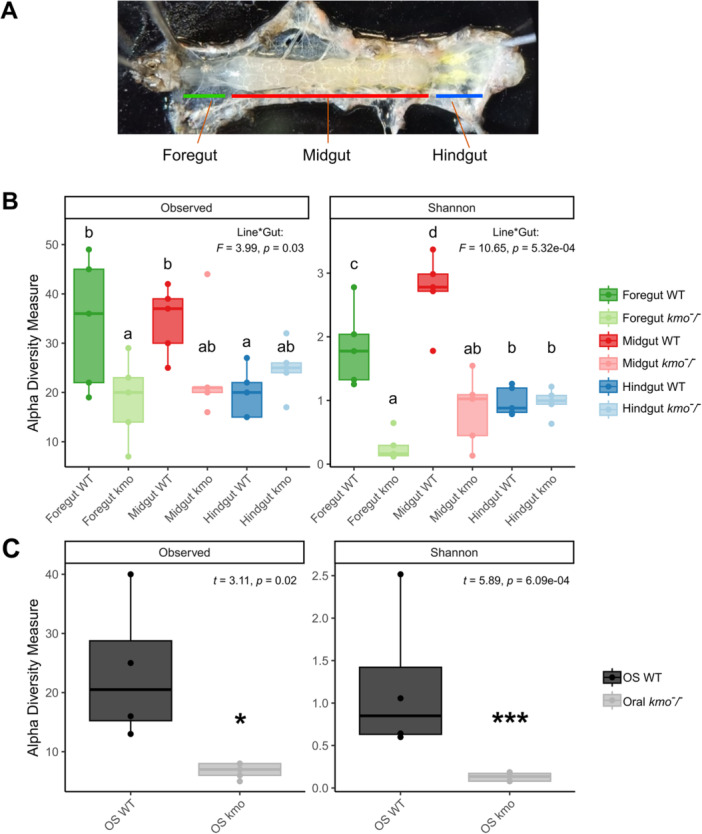
Effects of *kmo* knockout on bacterial alpha diversity in the digestive tract of *Spodoptera exigua*. (A) Scheme of the dissected gut segments. (B) ASV richness and Shannon index in foregut, midgut, and hindgut. (C) ASV richness and Shannon index in oral secretions (OS). Foregut is showed in green, midgut in red, hindgut in blue, and oral secretions in black. Darker colors indicate wild‐type (WT) larvae, whereas lighter colors indicate *kmo* ^*‐/‐*^ knockout individuals. In the boxplot, boxes represent the interquartile range (IQR), horizontal lines indicate medians, and whiskers extend to 1.5 × IQR. Statistical significance of 8‐HQA loss on alpha diversity (ANOVA or t‐test) is reported in the top left of each panel. Groups not sharing a letter are significantly different according to Fisher's least significant difference (LSD) test.

Wild‐type and *kmo* ^‐^/^‐^ larvae were reared side by side under identical environmental conditions until the fifth instar. Within 24 h after the fourth‐to‐fifth instar molting, the OS were collected under sterile conditions in a laminar flow hood and immediately frozen at ‐80°C. After ~2 h of recovery, larvae were cold‐anesthetized and pinned for dissection. The cuticle was opened ventrally and foregut, midgut, and hindgut were separated (Figure 2A). Each gut section was placed in a tube containing 300 μL of TRItidy G (PanReac AppliChem), a single‐phase reagent used for RNA extraction. Microbial communities were characterized by ribosomal 16S rRNA metabarcoding. For gut sections, total RNA was used to profile transcriptionally active bacteria, whereas DNA was used for OS due to the lower microbial abundance recovered from these samples. Consequently, gut and OS datasets were interpreted independently, and only qualitative concordance between them was considered. Each biological replicate consisted of pooled material from five individuals.

DNA extraction from OS was carried out by Microomics Systems (Barcelona, Spain) in controlled white room conditions. Total RNA was extracted following the TRItidy G manufacturer's protocol and resuspended in RNase‐free Milli‐Q water. RNA concentration was quantified using a NanoDrop spectrophotometer, and RNA integrity was verified on a 1% agarose gel. One microgram of purified RNA was treated with DNase I (Thermo Fisher Scientific) according to the manufacturer's instructions and subsequently used for cDNA synthesis using the PrimeScript RT Reagent Kit (Takara Bio Inc., Kusatsu, Japan). cDNA was sent to Microomics Systems for amplification of the V3–V4 regions, library preparation, and Illumina MiSeq paired‐end sequencing (2 ×300 bp; ~ 60,000–80,000 reads per sample). Amplification used primers SD‐Bact‐0341‐b‐S‐17 (5′‐CCTACGGGNGGCWGCAG‐3′) and SD‐Bact‐0785‐a‐A‐21 (5′‐GACTACHVGGGTATCTAATCC‐3′) (Herlemann et al. 2011), which target the V3‐V4 region of the 16 s rRNA gene. In total, 46 samples were sequenced: 40 from *S. exigua*, 4 negative controls, and 2 positive controls (ZymoBIOMICS mock community). Raw data are deposited in the NCBI Sequence Read Archive (SRA) under BioProject accession PRJNA1353060.

### 2.5 Quantification of Bacterial Abundance in Foregut and Midgut by qPCR

To obtain an independent estimate of bacterial abundance in gut tissues, foregut and midgut samples from fifth‐instar wild‐type and *kmo* ^‐^/^‐^ larvae were dissected as described above and pooled in pools of five individuals per biological replicate. Total RNA was extracted, treated with DNase I, and reverse‐transcribed into cDNA as described above. Bacterial abundance was then estimated by quantitative PCR (qPCR) targeting the 16S rRNA gene using the primer pair 16S_341dF (5'‐CCTACGGGNGGCWGCAG‐3') and 16S_806dR (5'‐GGACTACHVGGGTWTCTAATC‐3') with DNA from *Escherichia coli* used to generate a calibration curve. Amplification reactions were performed in a StepOnePlus Real‐Time PCR System (Applied Biosystems) in a final volume of 20 µL containing 5× HOT FIREPol EvaGreen qPCR Mix Plus (Solis BioDyne), 1 µL of template DNA, and 0.125 µM of each primer. This analysis was performed to compare the bacterial abundance in the two genotypes in gut tissues independently of the metabarcoding analysis.

### 2.6 Metabarcoding Library Filtering and Taxonomic Classification

Bioinformatic analyses were performed in R v4.5.0 (R Core Team 2018) following the DADA2 workflow (Callahan et al. 2016). Reads were trimmed with *filterAndTrim* (DADA2), using parameters selected with Figaro (Weinstein et al. 2019); primers were removed with Cutadapt (Martin 2011). Error modeling (*learnErrors*), read merging (*mergePairs*), and chimera removal (*removeBimeraDenovo*) were then performed in DADA2. Taxonomy was assigned using the Ribosomal Database Project (RDP‐II version 19; Cole et al. 2007)) using *assignTaxonomy* of DADA2. The mock community was processed following the Micro4all workflow (Wentzien 2023) with the function *MockCommunity*. Amplicon sequence variants (ASVs) accounting for < 0.005% of total reads were removed, as well as those classified as Eukaryota, mitochondria, or chloroplasts.

### 2.7 Biodiversity Analyses on Gut Bacterial Communities

The impact of 8‐HQA loss on bacterial communities was analyzed separately for gut (RNA‐based) and OS (DNA‐based). Rarefaction curves were generated with *ggrare* of ranacapa R package (Kandlikar et al. 2018). Alpha diversity‐related parameters were estimated using the function *estimate_richness* of library phyloseq (McMurdie and Holmes 2013), and their correlations with read depth were tested using Spearman's rank correlation (*cor.test*). Alpha diversity comparisons were performed with *t*‐tests for OS and ANOVA followed by Fisher's least significant difference (LSD) test for gut samples (*LSD.test* of package agricolae (Mendiburu 2023). Normality and homoscedasticity were assessed with shapiro.test and leveneTest (car package; Fox and Weisberg 2019); data were log‐transformed when necessary.

To minimize potential biases caused by uneven sequencing depth across samples, the data were normalized using the cumulative sum scaling (CSS) method prior to beta diversity analyses with *phyloseq_transform_css* of metagMisc package (Mikryukov 2017). Bray–Curtis dissimilarities were used for principal coordinate analyses (PCoA). Differences in bacterial community composition were assessed with PERMANOVA using *adonis2* function from vegan package (Oksanen et al. 2025). Group dispersion homogeneity was verified with *betadisper* (vegan) to ensure that differences in centroid positions reflected a real treatment impact on *S. exigua* bacteria without the influence of dispersion‐related effects. Then, differentially abundant taxa were identified using the R package microeco (Liu et al. 2021). Linear discriminant analysis Effect Size (LEfSe) was applied using the function *trans_diff* and taxa displaying a LDA score ≥ 3.5 were selected.

### 2.8 Comparison of Growth and Developmental Parameters in Wild‐Type and *kmo*⁻^/^⁻ Larvae

Total weight gain and developmental time of wild‐type and *kmo* ^‐^/^‐^ individuals during the larval stage were compared using either artificial diet or pepper leaves (*Capsicum annuum* var. Dulce de España) as feeding substrates. For each condition (wild‐type or *kmo* ^‐^/^‐^; artificial diet or pepper leaves), a minimum of 50 neonates were reared individually. Larvae fed on artificial diet were placed in 32‐well bioassay trays (Frontier Agricultural Sciences, US), whereas those fed on pepper leaves were maintained in 37 mL plastic cups (Frontier Agricultural Sciences, US). Individuals were supplied daily with fresh food according to their assigned diet. Rearing conditions were 25 ± 3°C, 60 ± 5% relative humidity, and a 16:8 (L:D) photoperiod. Developmental stages and mortality were recorded daily, and individuals were weighted daily from the fourth instar until pupation. Analyses and figures were performed in R v4.4.3 (R Core Team 2018). Differences in instar progression over time were analyzed separately for each diet using generalized linear models (GLMs) with a binomial error structure and logit link, including the genotype and day as fixed effects using glmmTMB (McGillycuddy et al. 2025) and DHARMa (Hartig 2025) packages. Time to pupation was further assessed by Kaplan‐Meier survival curves with log‐rank tests using survival (Therneau 2024) and survminer (Kassambara et al. 2025) packages. Maximum weight gain was defined as the highest weight recorded for each individual at any given time point, and differences between treatments were analyzed using unpaired *t*‐test with Welch's correction. At least two independent experiments were performed for each condition. All graphs were performed with ggplot2 or GraphPad Prism (v8.2.1).

### 2.9 Dietary Supplementation With 8‐HQA

To test whether the addition of 8‐HQA could rescue the growth impairment of the *kmo* ^‐^/^‐^ colony, feeding assays were conducted with an artificial diet supplemented with 8‐HQA. At least 100 neonates from each colony (wildtype or *kmo* ^‐^/^‐^) were individualized in 128‐well bioassay plates (Bio‐Ba‐128, Frontier Agricultural Sciences, US) and reared on artificial diet containing 10 mM 8‐HQA ( > 98% HPLC, Sigma‐Aldrich). The compound was dissolved in dH_2_O, adjusted to pH 10 with 0.5 M NaOH, and applied at 100 μL per well to the surface of the diet. Plates were allowed to dry before larvae were introduced. Control groups received only the solvent (dH_2_O, pH 10). Supplementation was freshly prepared and renewed every other day to ensure a constant stability and supply. Fitness traits (survival, developmental time, and total weight gain) were monitored as described above. To validate ingestion and persistence of supplemented 8‐HQA, an additional assay was performed using fourth‐instar *kmo* ^‐^/^‐^ larvae fed for 48 h on artificial diet containing 10 mM 8‐HQA. Control larvae received solvent only. After 48 h, OS were collected in pools of 16 larvae per biological replicate and analyzed by LC‐ESI‐MS/MS as previously described to confirm the detectability of 8HQA after diet supplementation.

### 2.10 Bioassays With *Bacillus thuringiensis* subsp. *aizawai*

The susceptibility of wild‐type and *kmo* ^*‐/‐*^ neonate larvae to *B. thuringiensis* subsp. *aizawai* was evaluated using the surface contamination method. Bioassays were conducted in 128‐well bioassay trays (Bio‐Ba‐128, Frontier Agricultural Sciences, US), containing artificial diet. As the *B. thuringiensis* subsp. *aizawai* source, the commercial formulation of Xentari (Kemnogard) was used. Seven concentrations were prepared in phosphate‐buffered saline (PBS), and in each replicate 16 larvae were tested per concentration. The tested range was established based on the previously reported LC_50_ of 1 ng/cm^2^ by Hernández‐Martínez et al. (2010). PBS alone served as the negative control. Mortality was recorded after 7 days. Each concentration was tested in at least three independent replicates. LC_50_ and LC_90_ values were estimated by Probit analysis (Finney 1971) with POLO‐PC (LeOra Software, US). Differences in LC₅₀ values were considered significant when their 95% fiducial limits (FL₉₅) did not overlap.

## 3 Results

### 3.1 Disruption of *kmo* by CRISPR‐Cas9 and Altered Coloration Phenotypes

In total, 425 eggs were injected with a mixture of gRNA targeting the third exon of the *kmo* gene and Cas9 enzyme (Figure 1A). From these, 56 individuals hatched (13.2%), and 26 developed into adult moths. Nine adults showed altered eye coloration compared to wild‐type individuals, corresponding to a successful editing rate of 16.1% among the hatched larvae, based on phenotypic changes. The *kmo* ^‐^/^‐^ population was established by intercrossing adults with the yellow‐eye phenotype (Figure 1B).

Beyond the change in compound eye color in adult moths, we observed additional phenotypic differences across developmental stages of *S. exigua* once the stable *kmo* ^‐^/^‐^ colony was established. Eggs appeared clearer and brighter, and larvae showed a lighter overall pigmentation compared to wild‐type individuals. Notably, the ocelli and brain tissues in larvae shifted from dark brown to a completely white/transparent appearance (Figure 1D,E). In addition, the wing spots in adults changed from dark brown to golden yellow (Figure 1C).

### 3.2 Absence of 8‐HQA in *kmo* ^‐^/^‐^ Oral Secretions

To determine the presence of 8‐HQA in the oral secretion of *S. exigua*, we first analyzed the fragmentation pattern of a commercial 8‐HQA standard (Figure 1F). The retention time was 3.14 min, and the selected SRM was *m/z* 188 → 144, with a collision energy of 10 eV. Using these SRM parameters, we identified two peaks in the oral secretion of wild‐type larvae, with retention times of 2.82 and 3.14 min (Figure 1G). The latter peak was absent in the oral secretions of *kmo* ^*‐/‐*^ individuals (Figure 1H), confirming that it corresponded to 8‐HQA and was not produced by the mutant larvae. The concentration of 8‐HQA in wild‐type oral secretion ranged from 0.1 to 1 µg/µL.

### 3.3 Impact of *kmo* Knockout on *S. exigua* Bacterial Diversity

Gut bacteria from different sections of the *S. exigua* larval digestive tract were compared between wild‐type and *kmo* ^*‐/‐*^ individuals to assess compartment‐specific changes associated with the mutation. It is worth noting that while gut sections were metabarcoded based on 16S rRNA transcripts, thus profiling transcriptionally active bacteria, OS samples were metabarcoded using 16S rRNA gene DNA. A total of 3,117,819 raw reads were generated from 16S rRNA gene sequencing, of which 2,627,685 high‐quality reads were retained after filtering and contaminant removal, resulting in 200 ASVs. Although the overall diversity was low, sequencing depth was sufficient to capture the *S. exigua* gut bacterial communities, as rarefaction curves reached saturation for all but one sample (Supporting Information S1: Figure 1). The excluded sample (OS from wild‐type larvae) yielded ~30‐fold fewer reads than the average of its compartment (OS of wild‐type larvae; Supporting Information S1: Table 1) and was therefore removed from downstream analyses. Consequently, 39 *S. exigua* samples were finally retained for further analysis.

Read depth was negatively correlated with alpha diversity metrics. For gut sections, this effect was only observed for the Shannon index, whereas in OS both ASV richness and Shannon diversity were negatively correlated with read number (Supporting Information S1: Figure 2). The *kmo* mutation significantly reduced ASV richness in the foregut and OS (*F* = 3.99, *p* = 0.03 and *t* = 3.11, *p* = 0.02, respectively, Figure 2). In addition, Shannon diversity was further reduced along the digestive tract, with significant effects also detected in the midgut (*F* = 10.65, *p* = 5.32e‐04 and *t* = 5.89, *p* = 6.09e‐04, respectively, Figure 2).

**Figure 3:**
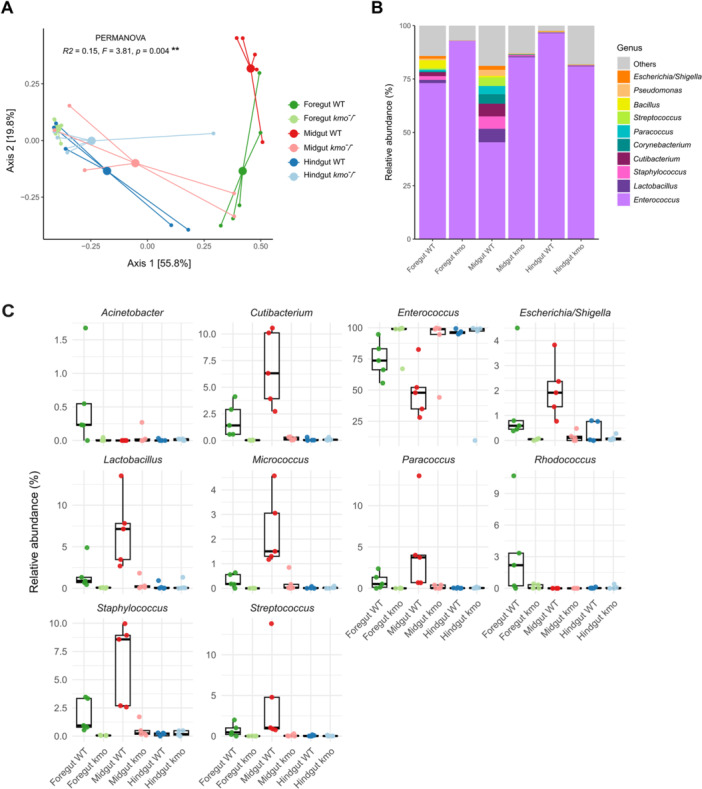
Effects of *kmo* knockout on bacterial community composition in different gut sections of *Spodoptera exigua*. (A) Principal Coordinates Analysis (PCoA) based on Bray–Curtis dissimilarity showing bacterial community structure across gut sections. Small dots represent individual samples; larger dots represent group centroids. The percentage of variance explained by each axis is shown in brackets. (B) Relative abundance of the top 10 bacterial genera. Genera with lower relative abundance were pooled into the “Others” category. (C) Genera contributing to compositional differences between gut sections according to Linear discriminant analysis Effect Size (LEfSe) with a LDA score ≥ 3.5. Foregut = green, midgut = red, hindgut = blue, oral secretions = black. Darker colors indicate wild‐type (WT) and lighter colors *kmo* ^*‐/‐*^ mutants.

The differences in sequenced read abundance between *kmo* ^*‐/‐*^ and wild‐type larvae were further validated by qPCR quantification of 16S rRNA transcripts using pooled foregut and midgut samples. This analysis revealed higher levels of 16S rRNA transcripts in *kmo* ^*‐/‐*^ larvae (Mann–Whitney test, *p* = 0.0007) (Supporting Information S1: Figure 3).

### 3.4 Impact of *kmo* Knockout on Bacterial Communities Across Gut Sections of *S. exigua*

The *kmo* mutation altered the bacterial composition at the genus level along the digestive tract of *S. exigua*. In wild‐type individuals, foregut, midgut and hindgut harbored distinct bacterial communities, whereas this compartmental differentiation was lost in *kmo* ^*‐/‐*^ larvae. In the mutant line, all gut sections displayed a similar bacterial composition, resembling that of the wild‐type hindgut (Figure 3, Supporting Information S1: Table S1. Significant differences between wild type and *kmo* ^*‐/‐*^ individuals were detected in the foregut and midgut, but not in the hindgut (Supporting Information S1: Table S2). Overall, the gut bacterial communities were dominated by the genus *Enterococcus*, which almost exclusively accounted for the hindgut microbiota of wild‐type larvae and for all gut sections of *kmo* ^*‐/‐*^ individuals. In contrast, the foregut and especially the midgut of wild‐type larvae showed higher relative abundances of additional genera besides *Enterococcus* (Figure 3B).

In wild‐type individuals, a major driver of compositional dissimilarities across gut sections was the relative abundance of *Enterococcus*, although several other genera contributed to specific differences (Figure 3B). For example, in the foregut‐midgut comparison, *Enterococcus*, *Rhodococcus* and *Acinetobacter* were more abundant in the foregut, whereas *Micrococcus*, *Cutibacterium* and *Lactobacillus* were enriched in the midgut (Figure 3C). The midgut also harbored higher abundances of *Staphylococcus, Streptococcus, Paracoccus* and *Escherichia/Shigella* (Figure 3C). In foregut‐hindgut comparison, apart from the higher representation of *Enterococcus* in the hindgut, the differences were mainly driven by *Cutibacterium, Staphylococcus* and *Lactobacillus* (Figure 3C).

When comparing individuals not expressing *kmo*, *Enterococcus* consistently emerged as the major driver of compositional shifts, being more abundant in the foregut and midgut of *kmo* ^*‐/‐*^ mutants (Figure 3C). The midgut showed the largest number of genera affected by the mutation: *Cutibacterium*, *Staphylococcus*, *Lactobacillus* and *Escherichia/Shigella* were consistently more abundant in wild‐type larvae than in *kmo* ^*‐/‐*^ mutants, both in foregut and midgut (Figure 3C). Additional genera such as *Streptococcus*, *Paracoccus* and *Micrococcus* were also enriched in the midgut of wild‐type individuals compared to mutants.

### 3.5 Impact of *kmo* Knockout on *S. exigua* OS Bacterial Composition

**Figure 4:**
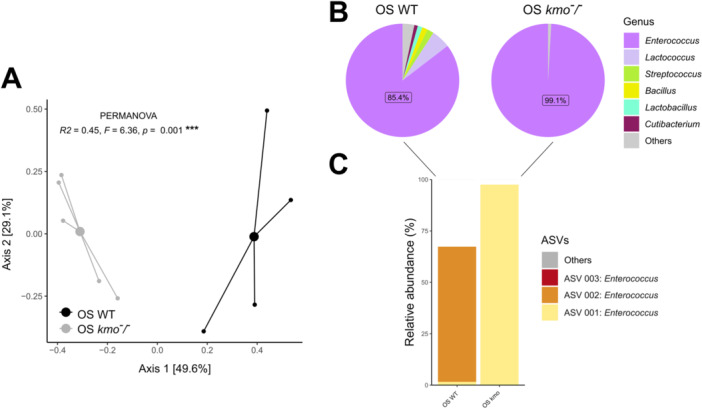
Effects of 8‐HQA loss in *Spodoptera exigua* oral secretion (OS) bacterial composition. (A) Principal Coordinates Analysis (PCoA) calculated with Bray‐Curtis dissimilarity. Small dots represent individual samples; larger dots represent group centroids. The percentage of variance explained by each axis is shown in brackets (B) Relative abundance of top *S. exigua* OS bacteria at the genus level. Low abundant taxa were merged into “Others”. (C) Relative abundance of top *S. exigua* OS bacteria at the amplicon sequence variant (ASV) level. WT: wild‐type.

Bacterial composition of the OS also differed between wild‐type and *kmo* ^‐^/^‐^ colonies (Figure 4A). This variation was primarily driven by changes in the relative abundance of *Enterococcus*, which was markedly higher in *kmo* ^*‐/‐*^ individuals compared to the wild‐type (Figure 4B). ASV‐level analysis revealed even more pronounced differences. In the *kmo* ^*‐/‐*^ colony, nearly all detected sequences corresponded to a single *Enterococcus* ASV, indicating a strong dominance of this taxon. In contrast, this ASV was almost absent in the wild‐type colony, where two alternative ASVs together accounted for up to 80% of the microbial abundance (Figure 4C). These results highlight substantial changes in bacterial community structure in OS, consistent with the gut alterations associated with the loss of 8‐HQA.

### 3.6 Impaired Larval Development and Survival of *kmo* ^‐^/^‐^ Mutants

**Figure 5:**
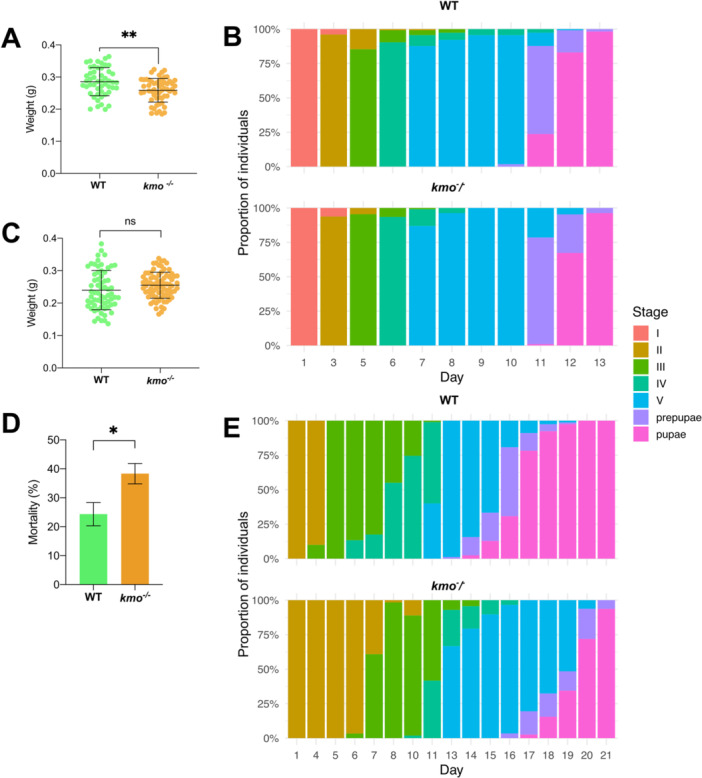
Fitness costs of *kmo* knockout in *Spodoptera exigua* larvae reared under artificial and pepper diets. (A) and (C) Maximum weight gained in larval stage on artificial and pepper leaf diets, respectively. (B) and (D) Proportion of developmental stages over time when fed on artificial and pepper leaf diets, respectively. (E) Total mortality observed during larval development on the pepper leaf diet.

To assess whether *kmo* disruption and the consequent changes in gut microbiota impose fitness costs during the larval stage of *S. exigua*, developmental parameters were compared between wild‐type and *kmo* ^*‐/‐*^ individuals. On artificial diet, *kmo* ^*‐/‐*^ larvae reached significantly lower maximum weights than wild‐type larvae (Figure 5A, unpaired *t*‐test, *t* = 3,380, *df* = 96.44, *p* = 0.001). When examining developmental timing across larval stages, only minor differences were found between genotypes in the second and fifth instars and in the prepupal stage, but not in the first, third, or fourth instar (Figure 5B, Supporting Information S1: Table S3). Similarly, survival analysis showed that time to pupation was affected by genotype (log‐rank test *χ* ^2^(1) = 19.5, *p* < 0.0001, Supporting Information S1: Figure 4), although the average duration from hatching to pupation remained around 13 days for both colonies. Even though these differences are subtle, they point to a fitness cost associated with the *kmo* mutation during larval development on an artificial diet.

**Figure 6:**
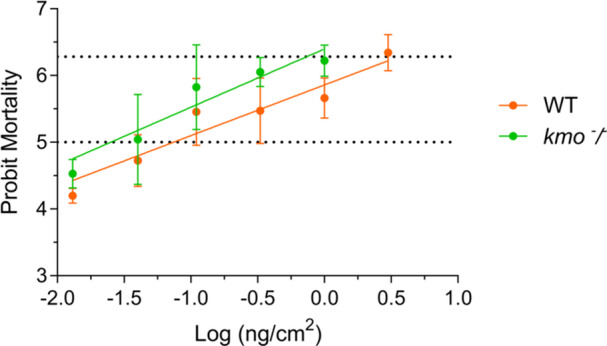
Mortality of wild‐type and *kmo*⁻/⁻ *Spodoptera exigua* larvae exposed to *Bacillus thuringiensis* subsp. *aizawai*. Dose–response curves of mortality induced by *B. thuringiensis var aizawai* (Xentari) against 1st instar wild‐type (WT) and *kmo* ^*‐/‐*^ larvae.

To test whether the reduced weight gain in *kmo* ^*‐/‐*^ larvae could be rescued by 8‐HQA, we supplemented the artificial diet with 10 mM 8‐HQA, a concentration exceeding natural gut levels (Pesek et al. 2015). A two‐way ANOVA (genotype x treatment) revealed a significant interaction (F_1,208_ = 7.01, *p* = 0.0087) (Supporting Information S1: Figure 5). Without supplementation, wild‐type larvae were heavier than *kmo* ^*‐/‐*^, as expected (Δ = 0.0266; Šidák‐adjusted; significant), but no difference was observed with 8‐HQA supplementation (Δ = −0.0023; n.s.). Within each colony, the effect of 8‐HQA supplementation was not significant (wild‐type: Δ = 0.0131; n.s.; *kmo* ^*‐/‐*^: Δ = −0.0158; n.s.). These results indicate that although 8‐HQA attenuated the weight difference between wild‐type and *kmo* ^⁻/⁻^ larvae observed in the control diet, it did not restore wild‐type values. To verify that supplemented 8‐HQA remained detectable after oral intake, we performed an additional validation assay in fourth‐instar *kmo* ^⁻/⁻^ larvae fed for 48 h on an 8‐HQA‐supplemented diet. LC‐ESI‐MS/MS analysis of oral secretions revealed a clear 8‐HQA peak in supplemented larvae, whereas solvent‐fed controls showed no detectable signal (Supporting Information S1: Figure 6). The estimated concentration of 8‐HQA in *kmo* ^⁻/⁻^ larvae fed on supplemented diet was 0.25 µg/µL.

When reared exclusively on fresh pepper leaves, both colonies reached comparable maximum weights (Figure 5C, unpaired t‐test, *t* = 1.715, *df* = 107.7, *p* = 0.08). Nonetheless, mortality was higher in *kmo* ^*‐/‐*^ larvae (38.3%) than in wild‐type (24.3%), indicating reduced adaptation to the plant diet (Figure 5D, unpaired t‐test *t* = 4.529, *df* = 4, *p* = 0.01). Moreover, *kmo* ^‐^/^‐^ larvae exhibited altered instar durations for all stages compared to wild‐type, with particularly prolonged second and fifth instars (Figure 5E, Supporting Information S1: Table 3). The *kmo* ^⁻/⁻^ genotype also had a significant, albeit smaller, effect on the prepupal stage (GLM, *p* = 0.012). Survival analysis further showed that time to pupation was strongly affected by genotype, with wild‐type larvae pupating significantly earlier (log‐rank test *χ* ^2^(1) = 93, *p* < 0.0001, Suppl. Fig. YB). Overall, total developmental time until pupation was delayed by more than 2 days in *kmo* ^⁻/⁻^ individuals compared to wild‐type.

### 3.7 *kmo* ^‐^/^‐^ Susceptibility to *Bacillus thuringiensis*

The susceptibility of the *kmo* ^⁻/⁻^ colony to *B. thuringiensis* (Bt), a bacterial entomopathogen frequently used to control *S. exigua*, was evaluated. Bioassays with neonate larvae from both populations were conducted using discriminating concentrations of Xentari (a *Bt*‐based commercial product). The *kmo* ^⁻/⁻^ colony was found to be approximately three times more susceptible to this compound than the wild‐type colony (Figure 6, Table 1). The estimated LC₅₀ for the *kmo* ^⁻/⁻^ colony (0.031 ng/cm²) was notably lower than that of the wild‐type strain (0.118 ng/cm²), and the non‐overlapping fiducial limits indicated a statistically significant difference in susceptibility. Similarly, LC₉₀ values followed the same trend, with *kmo* ^⁻/⁻^ individuals requiring roughly three times less toxin to achieve equivalent mortality. The slopes of the probit regressions were comparable between genotypes, indicating similar response homogeneity in both populations. Although these differences are moderate, the results suggest that disruption of the kynurenine pathway is associated with increased susceptibility to *B. thuringiensis*, in parallel with marked alterations in gut microbial homeostasis.

**Table 1:** Lethal concentration values (LC₅₀ and LC₉₀) estimated by probit analysis.

Colony	LC_50_ (FL_95%_)^a^	LC_90_ (FL_95%_)^a^	*n*	Slope ± SE^b^
Wild‐type	0.118 (0.051–0.214)	1.756 (1.094–3.018)	729	1.094 ± 0.116
*kmo* ^‐^/^‐^	0.031 (0.019–0.045)	0.515 (0.356–0.804)	872	1.052 ± 0.093

^a^ Toxin concentrations are expressed as ng/cm². FL Fiducial limits

^b^ SE standard error.

## 4 Discussion

The kynurenine 3‐monooxygenase (*kmo*) gene, also known as *cinnabar*, is a common CRISPR/Cas9 target in Lepidoptera because its disruption produces a visible pigmentation phenotype (W.‐K. Han et al. 2023; Hong et al. 2020; How et al. 2023; S.‐X. Ji et al. 2022; Mazumdar et al. 2023; Xu et al. 2020)*. kmo* ^⁻/⁻^ mutants usually show alterations in egg and adult eye pigmentation (W.‐K. Han et al. 2023; Hong et al. 2020; How et al. 2023; S.‐X. Ji et al. 2022; Xu et al. 2020). In our *S. exigua* mutants, we observed additional traits, including altered wing coloration and reduced pigmentation in ocelli, larval brain, and cuticle. Brain pigmentation by ommochromes was previously reported in *Bombyx mori* (Sawada et al. 2000) and *Plutella xylostella* (Xu et al. 2020), but its function remains unclear; one possibility is protection against UV radiation in transparent larval tissues. Wing color alterations were striking: in *Bicyclus anynana*, *kmo* knockout affected the eyes but not the wings (How et al. 2023), suggesting differences in ommochrome function between butterflies and moths.

Besides pigmentation, *kmo* disruption in *S. exigua* abolished 8‐HQA production, as in *S. littoralis* (Mazumdar et al. 2023). 8‐HQA is a quinoline carboxylic acid mainly produced by *Spodoptera* species, where it has been proposed to act as an iron‐chelating siderophore (Pesek et al. 2014). In *S. littoralis*, the absence of 8‐HQA reduced gut bacterial diversity (Mazumdar et al. 2023), a pattern we also observed in *S. exigua*. To extend these findings, we analyzed the microbiota composition across the three main gut segments.

Wild‐type larvae harbored ~200 ASVs, a relatively simple microbiota community consistent with previous reports in *S. exigua*, at least in terms of core taxa (Gao et al. 2019; 2020; Martínez‐Solís et al. 2020; Mugo‐Kamiri et al. 2024; Ramírez‐Serrano et al. 2024; Zhou et al. 2022), and likely reflects the use of artificial diet, which provides fewer bacterial sources than natural plant‐based diets (Martínez‐Solís et al. 2020; Mason et al. 2020; Mugo‐Kamiri et al. 2024; Gayatri Priya et al. 2012). This relatively low richness is also explained by the fact that only active bacteria were included in the study, eliminating dead or latent bacteria from the analysis (as reported by Mugo‐Kamiri et al. (2024)). Moreover, a clear compartmentalization was evident: midgut had the highest diversity, followed by foregut, and finally the hindgut. This matches the midgut's central role in digestion and its lack of a cuticular lining, which is present in foregut and hindgut and renewed at each molt (Engel and Moran 2013; Wu et al. 2016). Cuticle shedding may disrupt bacterial communities, explaining the lower variability in these sections. Interestingly, despite this turnover, fluorescently labeled bacteria were observed in both foregut and hindgut across instars in *S. littoralis* (Teh et al. 2016). Physiological parameters such as pH may also contribute, as foregut is highly alkaline, midgut moderately acidic, and hindgut close to neutral (Funke et al. 2008), creating distinct colonization niches.

In *kmo* ^*‐/‐*^ mutants, this segment structure collapsed. Bacterial diversity decreased and all segments became similar to the wild‐type hindgut, dominated by *Enterococcus*, consistent with the results in *S. littoralis* (Mazumdar et al. 2023). In this study, loss of 8‐HQA was proposed to increase iron availability in the gut, potentially altering redox balance and affecting fermentative bacteria (Parmanand et al. 2019). However, whether iron availability is the primary mechanism through which 8‐HQA influences microbiota composition remains unresolved. In addition, quantification of 16S rRNA transcripts in the foregut and midgut showed higher bacterial abundance in the mutants, supporting that loss of *kmo* is associated with an increase in bacterial load, again consistent with results in *S. littoralis* (Mazumdar et al. 2023). In mosquitoes, *kmo* knockout also caused midgut dysbiosis, even though 8‐HQA production is not confirmed in these species, suggesting a broader role of the kynurenine pathway in gut microbiota homeostasis beyond lepidopteran larvae (Bottino‐Rojas et al. 2022). Also, 16S rRNA gene metabarcoding of bacteria present in the *S. exigua* larva oral secretions mirrored the findings observed in gut sections. In these samples, 16S metabarcoding was not focused on characterizing transcriptionally active bacteria but instead included all bacteria present, and the results showed a decrease in bacterial diversity in *kmo* ^⁻/⁻^ mutants with a clear switch in dominant *Enterococcus* ASVs.

Our findings, along with those from Mazumdar et al. (2023), indicate that a functional kynurenine pathway is necessary to maintain *Enterococcus* homeostasis. In the absence of 8‐HQA, *Enterococcus* species expand. This was evident in the oral secretion of *kmo* ^*‐/‐*^ larvae, where a normally marginal *Enterococcus* ASV outcompeted the three ASVs usually present (two of them *Enterococcus*). The role of *Enterococcus* in caterpillar larvae is increasingly recognized, with reports pointing that this genus influences diverse phenotypic traits including insecticide detoxification, immunity, and dietary adaptation. In *S. littoralis*, *E. casseliflavus* supplies six essential amino acids and its dysregulation negatively affects development and survival (Di Lelio et al. 2023). In *S. frugiperda*, *E. casseliflavus* increased larval mortality on both poor‐ and rich‐yeast diets but showed opposite effects on pupal weight (Fu et al. 2025). Another isolate, FAW2‐1, derived from wild *S. frugiperda* gut contents, improved *S. frugiperda* larval performance in a diet‐dependent manner (B. Chen et al. 2022; Mason et al. 2022). These examples highlight that the impact of *Enterococcus* is strongly shaped by dietary context.

In our assays, diet also modulated the consequences of *kmo* disruption. On an artificial diet, mutant larvae showed only a slight reduction in maximum weight and a few changes in developmental time. By contrast, on pepper leaves—a more restrictive diet—mutants showed a marked increase in mortality together with delayed developmental time. Interestingly, surviving mutants reached maximum weights comparable to wild‐type individuals, suggesting that the phenotype may reflect a threshold‐like survival effect rather than a uniform impairment of growth. In this scenario, loss of *kmo* may impose a critical physiological constrain under plant‐based diets, causing a subset of larvae to fail under dietary stress, while surviving individuals retain the ability to complete development. Maintaining a stable *Enterococcus* community appears crucial for coping with restrictive plant diets, which often have low nitrogen and contain toxins and protease inhibitors. This diet‐dependent effect may explain why *kmo* mutants in *S. frugiperda* and *S. littoralis* showed minimal fitness impacts when raised on an artificial diet (W.‐K. Han et al. 2023; Mazumdar et al. 2023), as observed in *S. exigua*. Interestingly, supplementing the artificial diet with 8‐HQA did not fully rescue the weight phenotype, although 8‐HQA could be detected in the oral secretions of *kmo* ^⁻/⁻^larvae fed on an 8‐HQA–supplemented diet, at concentrations comparable to the lower range of 8‐HQA found in the oral secretions of wild‐type larvae. A similar outcome was reported *in S. littoralis*, where 8‐HQA supplementation did not restore the wild‐type bacterial diversity (Mazumdar et al. 2023). These results suggest that although 8‐HQA likely contributes to microbiota regulation, disruption of the kynurenine pathway may have additional physiological consequences that are not fully compensated by exogenous supplementation.

Besides their role in facilitating dietary utilization, some *Enterococcus* species can also be pathogenic. For example, *E. faecalis* and *E. faecium* harm *S. littoralis* (Tang et al. 2012), whereas FAW2‐1 benefits *S. frugiperda* but is antagonistic to *S. exigua* under the same conditions (Mason et al. 2022). In *S. littoralis*, *E. mundtii* can suppress opportunistic pathogens through its bacteriocin, mundticin (Shao et al. 2017). Maintenance of microbial homeostasis via products of the kynurenine pathway, such as 8‐HQA, may therefore be important to keep opportunistic pathogens at low levels. This balance may be especially relevant when larvae face additional challenges such as *B. thuringiensis* infection, which triggers septicemia via the resident microbiota (Broderick et al. 2006; Caccia et al. 2016).

In our study, *kmo* ^*‐/‐*^ larvae displayed both marked dysbiosis and a three‐fold increase in susceptibility to Bt intoxication, suggesting a causal link between the two phenomena. Indeed, gut microbiota strongly influences Bt activity in *S. exigua*, as larvae lacking microbiota are less susceptible (Y. Li et al. 2022). Oral Bt infection also led to a marked increase in bacterial load in the midgut and hemolymph, particularly *E. mundtii*, which can become an opportunistic pathogen in the hemolymph (Y. Ji et al. 2025). Similar effects are reported in *Chilo suppressalis*, *Ostrinia furnacalis*, and *P. xylostella* (G. Chen et al. 2024; S. Li et al. 2021; 2025), and in *P. xylostella*, *E. mundtii* entered the hemolymph and caused septic death (L. Li et al. 2025). Other opportunistic pathogens may also contribute to septicemia, besides *Enterococcus*, and microbiota effects on Bt susceptibility vary among host‐pathogen combinations. For example, in *G. mellonella*, axenic larvae were more vulnerable to oral Bt, whereas *E. mundtii* inhibited Bt growth in vitro (Upfold et al. 2023). However, we cannot exclude the possibility that the increased susceptibility to Bt may also result from physiological consequences of *kmo* disruption independent of changes in gut microbiota.

Our results suggest that gut bacterial homeostasis, particularly of *Enterococcus*, is fundamental for larval fitness under natural dietary conditions. The kynurenine pathway and 8‐HQA production appear to represent evolved mechanisms in *Spodoptera* larvae to shape this core microbiota. By stabilizing gut communities, larvae can better exploit host plants and withstand biotic stressors such as pathogens, ultimately enhancing their ecological performance and biological efficacy.

## Author Contributions

**Daniel Pinos:** conceptualization (supporting), investigation (lead), formal analysis (equal), writing – original draft (equal), writing – review and editing (equal). **Elena García Marin:** investigation (supporting), writing – original draft (supporting). **Beatriz Ramírez Serrano:** formal analysis (equal), writing – original draft (supporting). **Luis Benavent Albarracín:** investigation (supporting). **Jordi Gamir:** investigation (supporting), funding acquisition (equal). **Cristina M. Crava:** conceptualization (lead), writing – original draft (equal), writing – review and editing (equal), funding acquisition (equal), supervision (lead).

## Ethics Statement

The authors has nothing to report.

## Conflicts of Interest

The authors declare no conflicts of interest.

## Supporting information

Supporting File

## Data Availability

The datasets generated during the current study are available in the GenBank Sequence Archive Reads (SRA) repository with BioProject ID PRJNA1353060.
