# Supplementary material for: Maintenance of Gut Microbial Balance via the Kynurenine Pathway Improves Larval Performance and Resistance to Bacillus thuringiensis in Spodoptera exigua
Source: Microbiologyopen. 2026 Apr 16;15(2):e70289. doi: 10.1002/mbo3.70289 (PMC13086628; doi:10.1002/mbo3.70289)
Supplement: Supplementary file 1 — Supporting File [file MBO3-15-e70289-s001.pdf]

Supplementary Figures

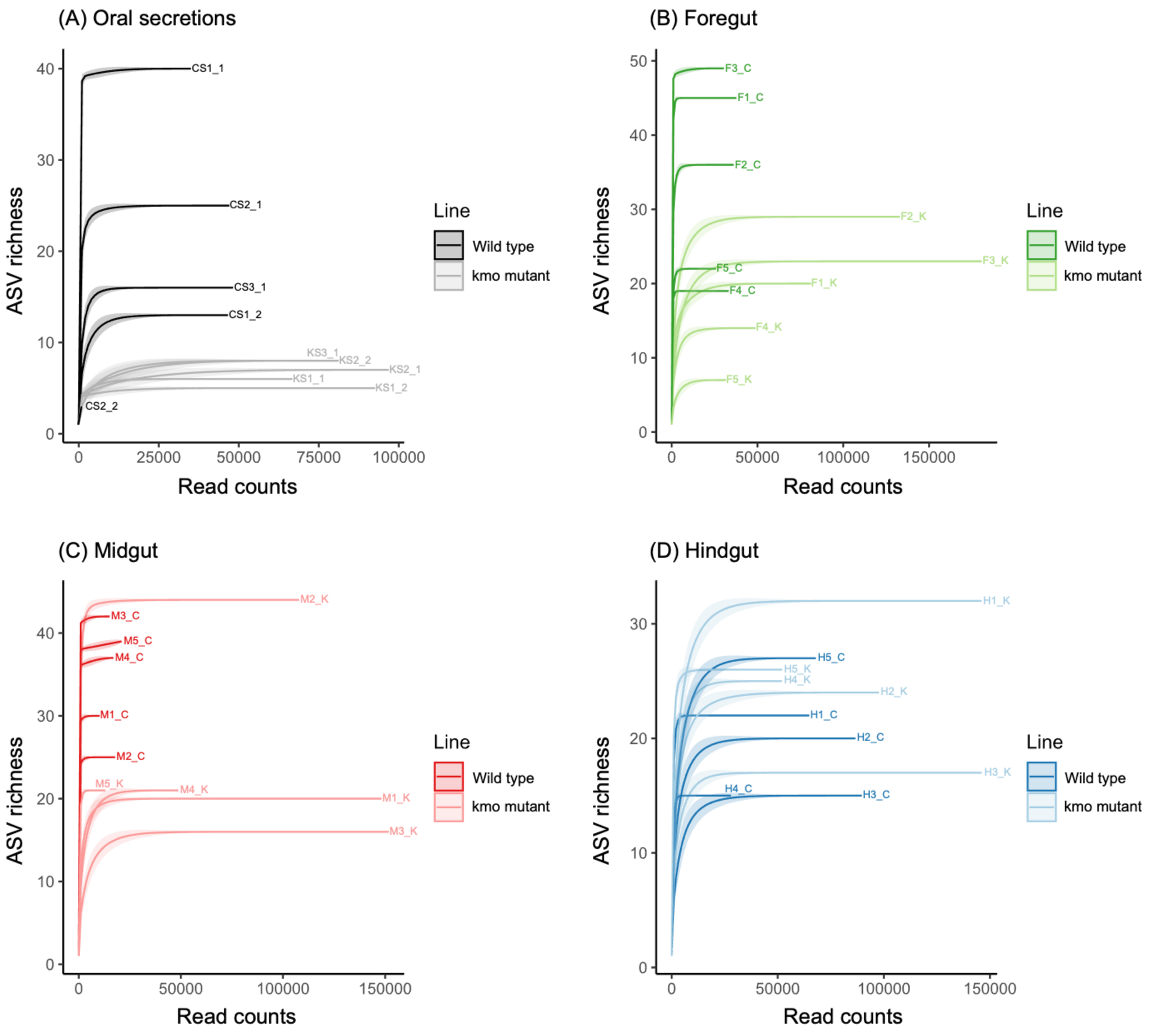

Figure S1. Rarefaction curves for *Spodoptera exigua* samples.
(A) Oral secretions, in black. **(B)** Foregut, in green. **(C)** Midgut, in red. **(D)** Hindgut, in blue. Dark colors represent wild type larvae and light color *kmo*$^{-/-}$ individuals.

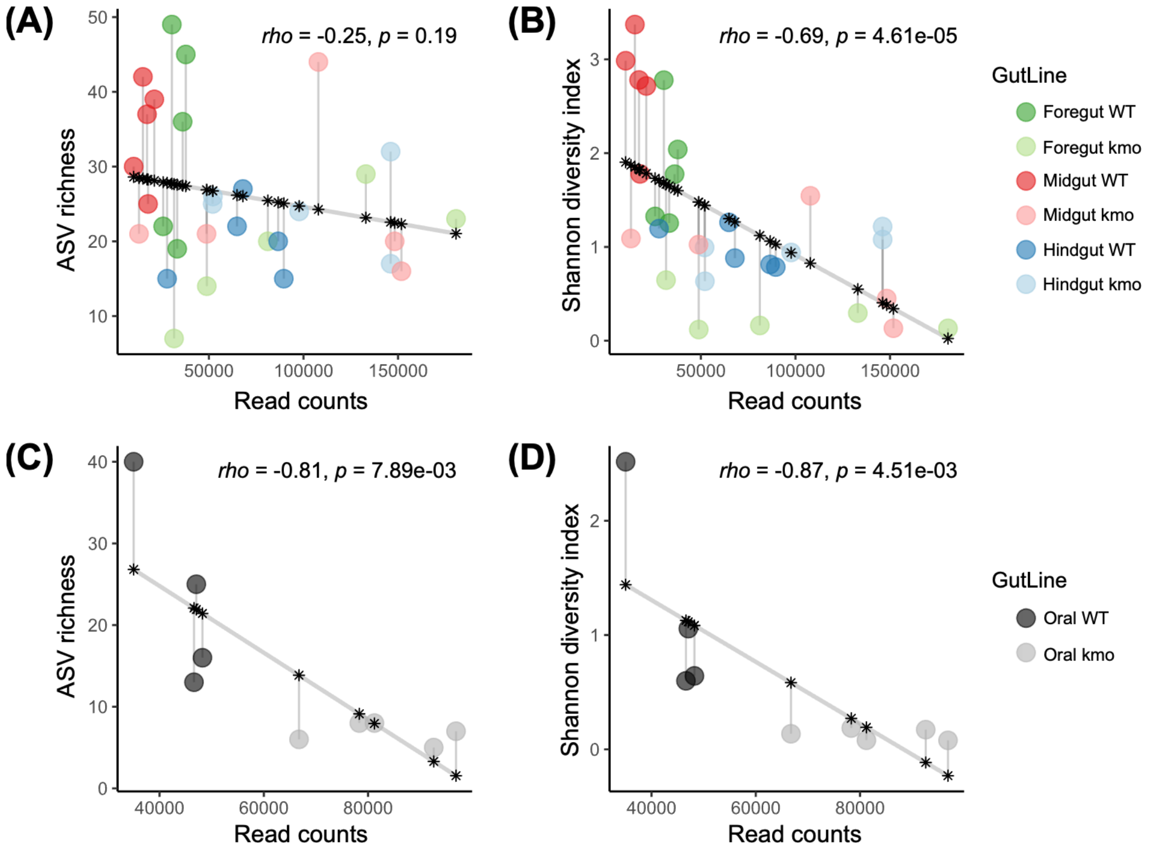

Figure S2. Correlation between ASV richness or Shannon diversity index and sample read counts.

Predicted values of the linear model are represented as asterisks and residuals as points. Foregut is represented in green, midgut in red, hindgut in blue and oral secretions in black. Dark colors represent wild type larvae and light color *kmo*$^{-/-}$ mutant individuals.

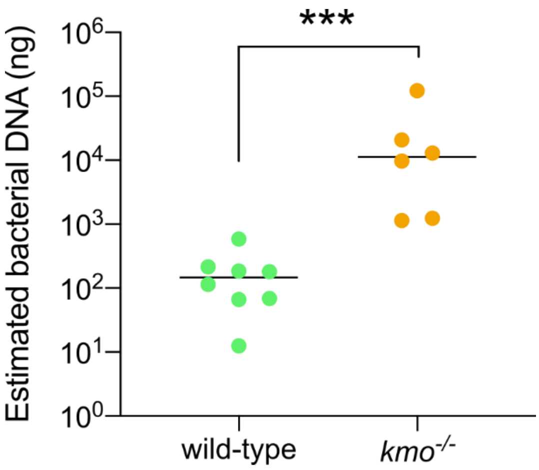

Figure S3. Estimation of bacterial abundance by qPCR.
The 16S rRNA gene was quantified from cDNA generated from total RNA, and relative bacterial abundance was estimated using a standard curve generated from known quantities of *E. coli* genomic DNA. Differences between groups were assessed using a Mann–Whitney test ($p = 0.0007$).

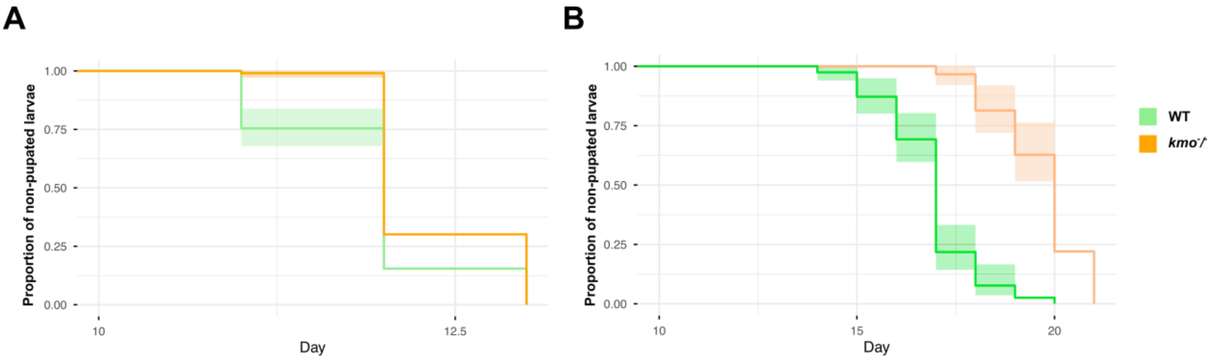

Figure S4. Kaplan-Meier survival curves of time to pupation. (A) Proportion of larvae without pupating in artificial diet. **(B)** Proportion of larvae without pupating in pepper leaf diet. Wild-type in green, *kmo*$^{-/-}$ mutants in orange.

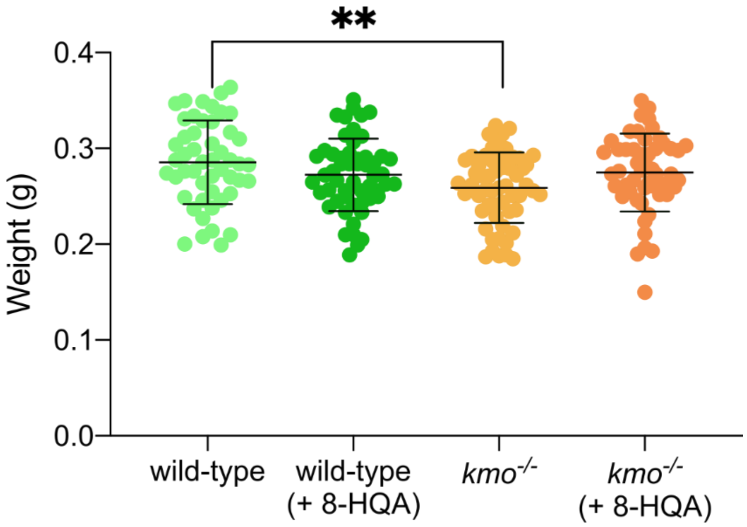

Figure S5. Effect of 8HQA (10 mM) supplementation on maximum weight gained in larval stage through surface contamination of artificial diet. Two-way ANOVA (genotype x treatment); significant interaction; Šidák post-hoc.

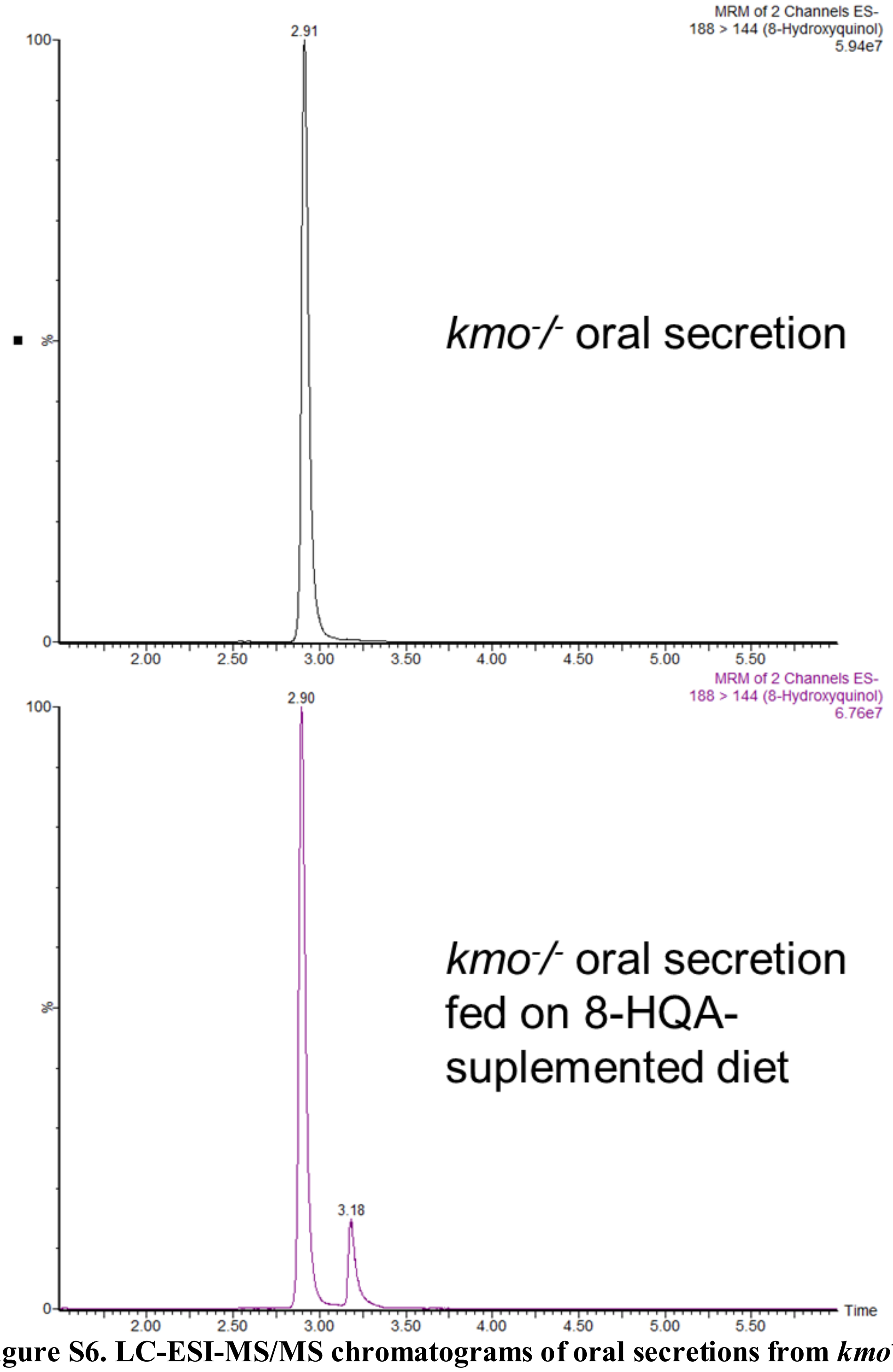

Figure S6. LC-ESI-MS/MS chromatograms of oral secretions from *kmo*$^{-/-}$ larvae fed on an unsupplemented diet or an 8-HQA–supplemented diet.

Fourth-instar larvae were fed for 48 h on diet supplemented with 8-HQA (lower panel) or on an unsupplemented diet (upper panel). The retention time of 8-HQA was 3.18 min**.**

Supplementary tables

Table S1. Sequencing information of samples across groups after filtering steps.

Sample	Line	Gut	GutLine	Reads	ASVs
CS1_1	Wild type	Oral secretions	OS WT	35036	40
CS1_2	Wild type	Oral secretions	OS WT	46606	13
CS2_1	Wild type	Oral secretions	OS WT	47053	25
CS2_2	*Wild type*	*Oral secretions*	*OS WT*	*1072*	*3*
CS3_1	Wild type	Oral secretions	OS WT	48225	16
F1_C	Wild type	Foregut	Foregut WT	37761	45
F2_C	Wild type	Foregut	Foregut WT	36110	36
F3_C	Wild type	Foregut	Foregut WT	30399	49
F4_C	Wild type	Foregut	Foregut WT	33225	19
F5_C	Wild type	Foregut	Foregut WT	25790	22
M1_C	Wild type	Midgut	Midgut WT	10203	30
M2_C	Wild type	Midgut	Midgut WT	17805	25
M3_C	Wild type	Midgut	Midgut WT	15098	42
M4_C	Wild type	Midgut	Midgut WT	17283	37
M5_C	Wild type	Midgut	Midgut WT	21123	39
H1_C	Wild type	Hindgut	Hindgut WT	64854	22
H2_C	Wild type	Hindgut	Hindgut WT	86608	20
H3_C	Wild type	Hindgut	Hindgut WT	89591	15
H4_C	Wild type	Hindgut	Hindgut WT	27887	15
H5_C	Wild type	Hindgut	Hindgut WT	67969	27
KS1_1	kmo mutant	Oral secretions	OS kmo	66750	6
KS1_2	kmo mutant	Oral secretions	OS kmo	92595	5
KS2_1	kmo mutant	Oral secretions	OS kmo	96868	7
KS2_2	kmo mutant	Oral secretions	OS kmo	81219	8
KS3_1	kmo mutant	Oral secretions	OS kmo	78315	8
F1_K	kmo mutant	Foregut	Foregut kmo	81160	20
F2_K	kmo mutant	Foregut	Foregut kmo	132987	29
F3_K	kmo mutant	Foregut	Foregut kmo	180662	23
F4_K	kmo mutant	Foregut	Foregut kmo	48868	14
F5_K	kmo mutant	Foregut	Foregut kmo	31571	7
M1_K	kmo mutant	Midgut	Midgut kmo	148253	20
M2_K	kmo mutant	Midgut	Midgut kmo	107877	44
M3_K	kmo mutant	Midgut	Midgut kmo	151788	16
M4_K	kmo mutant	Midgut	Midgut kmo	48736	21
M5_K	kmo mutant	Midgut	Midgut kmo	13012	21
H1_K	kmo mutant	Hindgut	Hindgut kmo	146083	32
H2_K	kmo mutant	Hindgut	Hindgut kmo	97711	24
H3_K	kmo mutant	Hindgut	Hindgut kmo	146100	17
H4_K	kmo mutant	Hindgut	Hindgut kmo	51963	25
H5_K	kmo mutant	Hindgut	Hindgut kmo	52116	26

OS: Oral secretions. WT: wild-type. In italic blue letters one sample displaying 30 times fewer read counts than its compartment average (oral secretions of wild-type individuals) that was discarded.

Table S2. Pairwise comparison of the bacterial composition of *Spodoptera exigua* along gut sections.

Comparison	*F*	*R2*	*p*
Foregut WT vs midgut WT	3.882	0.327	0.039
Foregut WT vs hindgut WT	5.997	0.428	0.011
Hindgut WT vs midgut WT	9.14	0.533	0.012
Foregut kmo vs hindgut kmo	1.042	0.115	0.53
Foregut kmo vs midgut kmo	2.338	0.226	0.122
Hindgut kmo vs midgut kmo	0.723	0.083	0.506
Foregut WT vs foregut kmo	20.707	0.721	0.009
Midgut WT vs midgut kmo	5.06	0.387	0.006
Hindgut WT vs hindgut kmo	0.61	0.07	0.63

Table S3. Generalized linear model p-values with genotype and day as fixed effects.

	stage	Factor	Df	Chisq	Pr(>Chisq)
artificial diet	I	genotype	1	0.687	0.4072
	I	day	10	1605.082	0
	II	genotype	1	8.027	0.0046
	II	day	10	1425.557	<0.0001
	III	genotype	1	0.237	0.6261
	III	day	10	1187.658	<0.0001
	IV	genotype	1	0.646	0.4215
	IV	day	10	1140.160	<0.0001
	V	genotype	1	13.044	0.0003
	V	day	10	2622.725	<0.0001
	prepupae	genotype	1	8.818	0.003
	prepupae	day	10	880.924	<0.0001
	pupae	genotype	1	29.030	<0.0001
	pupae	day	10	1773.591	<0.0001
pepper diet	I	genotype	1	-2.838E-13	1
	I	day	16	1.872E-11	1
	II	genotype	1	7.435E+02	<0.0001
	II	day	16	3.024E+03	<0.0001
	III	genotype	1	1.129E+01	0.0008
	III	day	16	1.628E+03	<0.0001
	IV	genotype	1	9.780E+01	<0.0001
	IV	day	16	7.430E+02	<0.0001
	V	genotype	1	8.998E+01	<0.0001
	V	day	16	1.933E+03	<0.0001
	prepupae	genotype	1	6.372E+00	0.01159
	prepupae	day	16	3.192E+02	<0.0001
	pupae	genotype	1	3.801E+02	<0.0001
	pupae	day	16	2.187E+03	<0.0001
